# Spirometric and anthropometric improvements in response to elexacaftor/tezacaftor/ivacaftor depending on age and lung disease severity

**DOI:** 10.3389/fphar.2023.1171544

**Published:** 2023-07-04

**Authors:** Katharina Schütz, Sophia Theres Pallenberg, Julia Kontsendorn, David DeLuca, Cinja Sukdolak, Rebecca Minso, Tina Büttner, Martin Wetzke, Christian Dopfer, Annette Sauer-Heilborn, Felix C. Ringshausen, Sibylle Junge, Burkhard Tümmler, Gesine Hansen, Anna-Maria Dittrich

**Affiliations:** ^1^ Department of Pediatric Pneumology, Allergology and Neonatology, Hannover Medical School, Hannover, Germany; ^2^ Biomedical Research in Endstage and Obstructive Lung Disease Hannover (BREATH), German Center for Lung Research, Hannover Medical School, Hannover, Germany; ^3^ Department of Respiratory Medicine, Hannover Medical School, Hannover, Germany; ^4^ European Reference Network on Rare and Complex Respiratory Diseases (ERN-LUNG), Frankfurt, Germany

**Keywords:** cystic fibrosis, BMI, elexacaftor/tezacaftor/ivacaftor, modulator, pediatric

## Abstract

**Introduction:** Triple-combination cystic fibrosis transmembrane conductance regulator (CFTR) modulator therapy with elexacaftor/tezacaftor/ivacaftor (ETI) was introduced in August 2020 in Germany for people with CF (pwCF) ≥12 years (yrs.) of age and in June 2021 for pwCF ≥6 yrs of age. In this single-center study, we analyzed longitudinal data on the percent-predicted forced expiratory volume (ppFEV1) and body-mass-index (BMI) for 12 months (mo.) after initiation of ETI by linear mixed models and regression analyses to identify age- and severity-dependent determinants of response to ETI.

**Methods:** We obtained data on 42 children ≥6–11 yrs, 41 adolescents ≥12–17 yrs, and 143 adults by spirometry and anthropometry prior to ETI, and 3 and 12 mo. after ETI initiation. Data were stratified by the age group and further sub-divided into age-specific ppFEV1 impairment. To achieve this, the age strata were divided into three groups, each according to their baseline ppFEV1: lowest 25%, middle 50%, and top 25% of ppFEV1.

**Results:** Adolescents and children with more severe lung disease prior to ETI (within the lowest 25% of age-specific ppFEV1) showed higher improvements in lung function than adults in this severity group (+18.5 vs. +7.5; *p* = 0.002 after 3 mo. and +13.8 vs. +7.2; *p* = 0.012 after 12 mo. of ETI therapy for ≥12–17 years and +19.8 vs. +7.5; *p* = 0.007 after 3 mo. for children ≥6–11 yrs). In all age groups, participants with more severe lung disease showed higher BMI gains than those with medium or good lung function (within the middle 50% or top 25% of age-specific ppFEV1). Regression analyses identified age as a predictive factor for FEV1 increase at 3 mo. after ETI initiation, and age and ppFEV1 at ETI initiation as predictive factors for FEV1 increase 12 mo. after ETI initiation.

**Discussion:** We report initial data, which suggest that clinical response toward ETI depends on age and lung disease severity prior to ETI initiation, which argue for early initiation of ETI.

## Introduction

The advent of highly effective cystic fibrosis transmembrane conductance regulator (CFTR) modulator therapy has led to an unprecedented improvement in the lives of people with CF (pwCF). Licensing of triple-combination CFTR modulator therapy in the form of elexacaftor (ELX)/tezacaftor (TEZ)/ivacaftor (IVA), henceforth termed ETI, increased the availability of CFTR modulator therapy. In Germany, ETI was introduced in August 2020 for pwCF ages 12 and older (pwCF ≥12), and in June 2021 for ages 6 and older (pwCF ≥6), permitting CFTR modulator therapy for more than 85% of patients in those age strata. Until now, determinants of ETI response have not been identified. Previous studies on other CFTR modulators suggest that age at therapy initiation significantly impacts upon response to CFTR modulator therapy.

Younger age at initiation of IVA permits better preservation of lung function and a larger impact on pulmonary exacerbation (PEx) (Bui et al., 2021). In adolescents, younger age at initiation is associated with a better percent-predicted forced expiratory volume (ppFEV1) response to LUM/IVA (Nichols et al., 2022), and adolescents have shown more favorable body-mass-index (BMI) trajectories than adults in response to LUM/IVA in a large real-world setting (Muilwijk et al., 2022). Similarly, the improvements in pancreatic function observed in very young patients initiating IVA or LUM/IVA also suggest that age at initiation of CFTR modulator therapy has an impact on specific organ functions (Davies et al., 2021; Rosenfeld et al., 2018; Merlo et al., 2022; McNamara et al., 2019).

By proxy, the findings of Nichols et al., which show a significant correlation between sweat chloride reductions and ppFEV1 improvements after 6 months of therapy (Nichols et al., 2022), might also suggest that ppFEV1 response to ETI is age-dependent since improvement in sweat chloride in response to CFTR modulators other than ETI has been observed to be larger when pwCF are younger (Durmowicz et al., 2013). With regard to improvement in CFTR function in response to ETI, phase III trials showed an improvement between −45 and −49 mmol/L for the two eligible genotype combinations in adolescents ≥12 years and adults (Heijerman et al., 2019; Middleton et al., 2019). Children aged 6–11 years of both eligible genotype combinations showed an overall improvement of −61 mmol/L sweat chloride (Zemanick et al., 2021), suggesting the response to ETI also shows an age dependency with regard to the magnitude of functional CFTR improvement. However, clinical data concerning longitudinal evolvement of pulmonary function and anthropometry, supporting similar age- or severity-dependent effects, are lacking for ETI.

Our study aimed to address determinants of response to ETI through the analysis of 3-month and 1-year follow-up (FU) data from a single-center cohort of *n* = 226 people with CF ≥ 6 years of age. We focused on comparing age strata with the similar severity of lung disease prior to ETI initiation, hypothesizing that earlier ETI initiation confers superior benefits with regard to the key clinical endpoints ppFEV1 and BMI.

## Materials and methods

### Study group, data collection, and study parameters

PwCF receive standardized diagnostic procedures and treatment in our center, according to national and international guidelines, including 3-month intervals of outpatient visits. All data used for this study were recorded in an in-house electronic patient record at every visit, according to definitions predefined by the German CF Registry.

Parameters from the last outpatient visit prior to ETI initiation were obtained for baseline values, including ppFEV1, BMI, sweat chloride, prior CFTR modulator use, sex, and age at ETI initiation. Values from inpatient visits were excluded from the analysis. Sweat chloride levels were determined prior to and 3 months after ETI initiation. The changes in ppFEV1 and BMI (in kg/m^2^) were collected prior to and on months 3, 6, 9, and 12 after ETI initiation. Lung function was referenced according to the Global Lung Function Initiative (GLI) (Quanjer et al., 2012). The BMI in kg/m^2^ was calculated electronically by weight and height measurements at every visit.

### Study design

We performed an exploratory, retrospective, single-center, post-approval cohort study. We extracted demographic data and data on ppFEV1 and BMI of all pwCF from our CF center, who received ETI therapy for at least 3 (for pwCF ≥6–11) or 12 (for pwCF ≥12) uninterrupted months. Data for pwCF ≥12 years were collected between August 2020 and January 2023, and data for pwCF ≥6, between June 2021 and January 2023. For patients ≥6–11 years of age, we only included data from FU 3 months in our analyses as the group size of further FU data was increasingly limited due to the recent approval of ETI in this age group.

Data were stratified according to age (≥6–11 yrs; ≥12–17 yrs; ≥18 yrs) and age-group-specific lung function impairment percentiles. We aimed to compare pwCF with similar degrees of lung disease across age groups. Yet, average ppFEV1 values, as a proxy for lung disease severity, are different across these age groups. We, therefore, chose to stratify pwCF according to the average ppFEV1 within their age group as reference, creating age-specific sub-groups of lung disease severity. For this purpose, we chose the common cut-off values of the lowest and highest 25th percentile for baseline ppFEV1 of the age group in question, thereby creating three groups: “severely affected”: baseline ppFEV1 ≤25th percentile (≤P25), “average”: 26th–74th percentile (P50), and “less affected”: ≥75th percentile (≥P75) for the age groups ≥6–11 yrs, ≥12–17 yrs, and ≥18 yrs, respectively. The age-dependent percentiles for ppFEV1 for our cohort were calculated and are also stated in Table 2.

• pwCF ≥18 years: ≤P25: 27.2 (23.4–30.4), P50: 53.1 (45.4–60.7), and
• >P75: 82.3 (77.0–92.9) for median (IQR)
• pwCF ≥12–17 years: ≤P25: 56.4 (50.2–61.9), P50: 83.4 (73.9–91.3), and
• >P75: 98.9 (95.5–110.9) for median (IQR)
• pwCF ≥6–11 years: ≤P25: 70.7 (61.4–80.7) P50: 87.7 (82.9–95.0), and
• >P75: 102.1 (100.6–105.4) for median (IQR)

### Statistical analysis

All statistical analyses were performed with Statistical Package for Social Sciences (SPSS 28, IBM, Armonk, New York, United States). Descriptive data were calculated as the median and interquartile range (IQR)**.** Initially, measurements were tested for normal distribution. Differences between groups were analyzed by the Mann–Whitney U test or Kruskal–Wallis test, as appropriate. Frequency differences between nominally distributed groups were calculated by a chi-squared test. Data were corrected for multiple testing by Bonferroni correction.

We used a generalized linear model to assess the effects of gender, age group, and prior CFTR modulator therapy on the outcome ppFEV1 or BMI for all pwCF at or above 12 years of age included in our analyses.

We performed regression analyses to address the effects of age, gender, and CFTR modulator therapy prior to ETI initiation, as well as baseline values for ppFEV1, BMI, and the severity of lung disease according to the previously defined percentiles at baseline. PpFEV1 or BMI gains at 3 or 12 months were considered outcomes. We included data from all pwCF at or above 6 years of age for gains at 3 months and from all pwCF above 12 years of age for gains at 12 months.

A *p*-value <0.05 was considered statistically significant.

### Ethics

All patients or their legal guardians provided consent to the anonymized scientific use of personal clinical data for research purposes, either as written informed consent to participate at scientific studies of the German Center for Lung Research (DZL) registry (Ethics Committee Hannover Medical School, #2923-2015, Hannover Medical School) and/or the German CF registry (Ethics Committee of the Justus-Liebig-Universität FB Medizin, #AZ24/19).

## Results

### Clinical characteristics of the entire study population

We were able to include *n* = 226 pwCF from our center into our analyses. Data were obtained at a median time of 3.1 months for all *n* = 226 subjects included in the study and 12.4 months for *n* = 182 (80.5%) subjects ≥12 years after ETI initiation. Median age at ETI initiation was 22.5 yrs (IQR 13.4–30.5), of which 51.8% were female subjects, and 45.1% already underwent CFTR modulator therapy prior to ETI initiation.

The median gain of ppFEV1 at 3 months FU was +9.7 (IQR 5.1–17.8) and +9.6 (IQR 5.5–16.4) at 12 months compared to ppFEV1 at baseline. The median BMI gain at 3 months FU was +0.8 kg/m^2^ (IQR 0.2–1.5) and +1.4 kg/m^2^ (IQR 0.4–2.6) at 12 months compared to prior ETI initiation (Table 1, Column 1). As expected, sweat chloride decreased by 44 mmol/L after 3 months of ETI therapy with no statistical differences in sweat chloride at baseline or after 3 months of ETI therapy between the three age groups (Table 1).

**TABLE 1:** Patients’ characteristics of the total cohort and stratified into three age groups (≥18 yrs, ≥6–11 yrs, and ≥12–17 yrs). Prior ETI therapy and F508del homozygosity were distributed differently between the three age groups. PpFEV1 and BMI at ETI initiation, and after 3 and 12 months of ETI therapy showed significant age-dependent differences, as did BMI gains at 3 months but not after 12 months. PpFEV1 gains or sweat chloride changes at all time points measured did not show age-dependent differences. *p*-values refer to Kruskal–Wallis or Mann–Whitney U inter-group comparisons for continuous variables and chi-squared tests for discontinuous variables.

Characteristic	All (*n* = 226)	*p*-value	≥18 years (*n* = 143)	≥12–17 years (*n* = 41)	≥6–11 years (*n* = 42)
Age at start (yrs), median (IQR)	22.5 (13.4–30.5)	0.000	27.5 (22.9–37.1)	13.8 (12.7–15.7)	9.3 (7.0–10.5)
Sex (female, %)	51.8	0.135	49.0	65.9	47.6
CFTR_prior ETI (%)	45.1	0.001	50.3	19.5	52.4
F508del homozygous (%)	54.4	0.013	60.1	34.1	54.8
FEV1% at initiation, median (IQR)	66.3 (45.7–84.6) (*n* = 226)	0.001	53.1 (33.9–71.1) (*n* = 143)	83.4 (66.6–95.4) (*n* = 41)	87.7 (79.7–97.5) (*n* = 42)
FEV1% at FU 3 months, median (IQR)	82.6 (57.4–99.2) (*n* = 226)	0.001	64.1 (44.7–87.6) (*n* = 143)	95.8 (80.5–107.2) (*n* = 41)	99.2 (88.2–106.0) (*n* = 42)
FEV1% at FU 12 months, median (IQR)	71.8 (52.5–93.0) (*n* = 182)	0.001	62.3 (46.0–87.2) (*n* = 143)	96.0 (78.2–107.4) (*n* = 39)	------
∆ FEV1% _(FU 3 mo.–start)_, median (IQR)	9.7 (5.1–17.8) (*n* = 226)	0.269	10.0 (5.3–18.0) (*n* = 143)	11.0 (5.2–19.1) (*n* = 41)	7.8 (3.2–14.9) (*n* = 42)
∆ FEV1% _(FU 12 mo.-start)_, median (IQR)	9.6 (5.5–16.4) (*n* = 182)	0.351	9.2 (5.4–16.6) (*n* = 143)	11.1 (7.1–14.9) (*n* = 39)	------
BMI (kg/m^2^) at initiation, median (IQR)	19.5 (17.3–21.5) (n = 226)	0.001	20.9 (18.9–22.4) (n = 143)	18.4 (17.1–20.3) (n = 41)	15.5 (14.7–16.4) (n = 42)
BMI (kg/m^2^) at FU 3 months, median (IQR)	20.6 (18.3–22.7) (*n* = 226)	0.001	21.6 (19.8–23.8) (*n* = 143)	19.3 (17.8–21.6) (*n* = 41)	15.7 (14.8–16.8) (*n* = 42)
BMI (kg/m^2^) at FU 12 months, median (IQR)	21.6 (19.4–23.9) (*n* = 182)	0.001	22.2 (19.8–24.2) (*n* = 143)	19.4 (18.1–21.7) (*n* = 39)	------
∆ BMI (kg/m^2^) _(FU 3 mo.–start)_, median (IQR)	0.8 (0.2–1.5) (*n* = 226)	0.001	0.9 (0.4–1.7) (*n* = 143)	1.2 (0.3–1.8) (*n* = 41)	0.3 (−0.2-0.6) (*n* = 42)
∆ BMI (kg/m^2^) _(FU 12 mo.–start)_, median (IQR)	1.4 (0.4–2.6) (*n* = 182)	0.869	1.4 (0.3–2.6) (*n* = 143)	1.3 (0.4–2.6) (*n* = 39)	-----
Chloride (mmol/L) at initiation, median (IQR)	95.0 (84.0–103.0) (n = 214)	0.495	95.0 (84.0–101.0) (n = 135)	98.0 (88.0–104.8) (n = 40)	93.0 (79.0–105.0) (n = 39)
Chloride (mmol/L) at FU 3 months, median (IQR)	44.0 (31.0–55.5) (*n* = 195)	0.890	44.0 (31.0–55.3) (n = 134)	42.5 (31.0–62.3) (*n* = 36)	45.0 (36.0–55.0) (*n* = 25)
∆ Chloride (mmol/L) _(FU 3mo–start)_, median (IQR)	47.5 (36.0–62.0) (*n* = 188)	0.789	49.0 (36.0–61.8) (*n* = 128)	47.5 (34.5–67.3) (*n* = 36)	45.5 (39.0–59.5) (*n* = 24)

### Age dependency of ppFEV1 and BMI response to ETI

To query age dependency of spirometric and anthropometric responses to ETI, we first stratified our cohort into participants ≥12–17 yrs of age (*n* = 41) and participants ≥18 yrs of age (*n* = 143) (Table 1).

Participants ≤18 yrs of age included a higher proportion of female subjects and used CFTR modulator therapy significantly less prior to ETI initiation. As expected, these patients had a higher ppFEV1 but lower absolute BMI values than participants ≥18 years of age. PpFEV1 at 3 and 12 months of ETI treatment were also significantly higher (*p* = 0.001), and BMI absolute values were still significantly lower in the group ≥12–17 yrs of age (*p* = 0.001).

Assessing predictors for the outcomes of absolute ppFEV1 or BMI after 12 months of ETI therapy, we found that neither gender nor prior CFTR modulator therapy influenced absolute values of ppFEV1 or BMI. It was only age at ETI initiation, which continued to exert an impact upon these absolute values, similar to the age dependency observed between the age groups at baseline.

Regression analyses for ppFEV1 gains revealed that after 3 months of ETI therapy, age and ppFEV1 at ETI initiation were significantly associated with ppFEV1 gains (*p* < 0.001 and *p* = 0.004, respectively), while neither gender, prior CFTR modulator therapy, nor BMI or degree of lung disease severity at baseline was associated with this outcome. BMI gains at 3 months were only influenced by ppFEV1 at baseline (*p* = 0.008), but none of the other parameters were tested.

Our analyses with only those patients aged 12 yrs and older showed similar trends with regard to the impact of age and ppFEV1 at ETI initiation upon ppFEV1 gains at 12 months after ETI initiation (*p* = 0.001 and *p* = 0.05, respectively). For BMI gains at 12 months, age at ETI initiation did have a significant effect (*p* = 0.004) as did BMI at initiation (*p* = 0.039), but contrary to the 3-month data, ppFEV1 at initiation did not associate with BMI gains.

Regression analyses of only those patients in the group with the most severe lung disease (ppFEV ≤P25, age ≥6-≥18 years, *n* = 55,) provided more insights into the critical role of age at initiation of ETI. Younger age is associated with significantly higher ppFEV1 gains at 3 and 12 months after ETI initiation (*p* < 0.001 and *p* < 0.002, respectively). Neither gender, prior CFTR modulator therapy, nor BMI or degree of lung disease severity at baseline was associated with ppFEV1 gains at 3 or 12 months.

### Severity of pre-existing lung disease does not impact upon ppFEV1 but on BMI response in the overall cohort

Given our results on age as a significant determinant of ppFEV1 and BMI gains, we hypothesized that pre-existing lung disease might exert different influences on ppFEV1 or BMI gains, depending on the age group analyzed. We, thus, stratified our cohort for age-specific severity of pre-existing lung disease by calculating age group-specific lung function percentiles (age-specific ppFEV1 ≤25th percentile (≤P25), 26th–74th percentile (P50), and ≥75th percentile (≥P75). This stratification did not lead to statistically significant differences with regard to age, proportion of female subjects, or prior CFTR modulator use (Supplementary Table S1). PpFEV1 gains at 3- and 12-month FU were similar in these three groups (8.1 (4.4–19.1) vs. 10.9 (5.7–19.9) vs. 9.3 (3.4–15.1) for 3-month FU (*p* = 0.209) and 9.0 (4.6–14.2) vs. 10.5 (5.6–22.0) vs. 9.2 (5.9–14.2) for 12-month FU (*p* = 0.258) for ppFEV1 ≤P25 vs. P50 vs. ≥P75, respectively.

**FIGURE 1:**
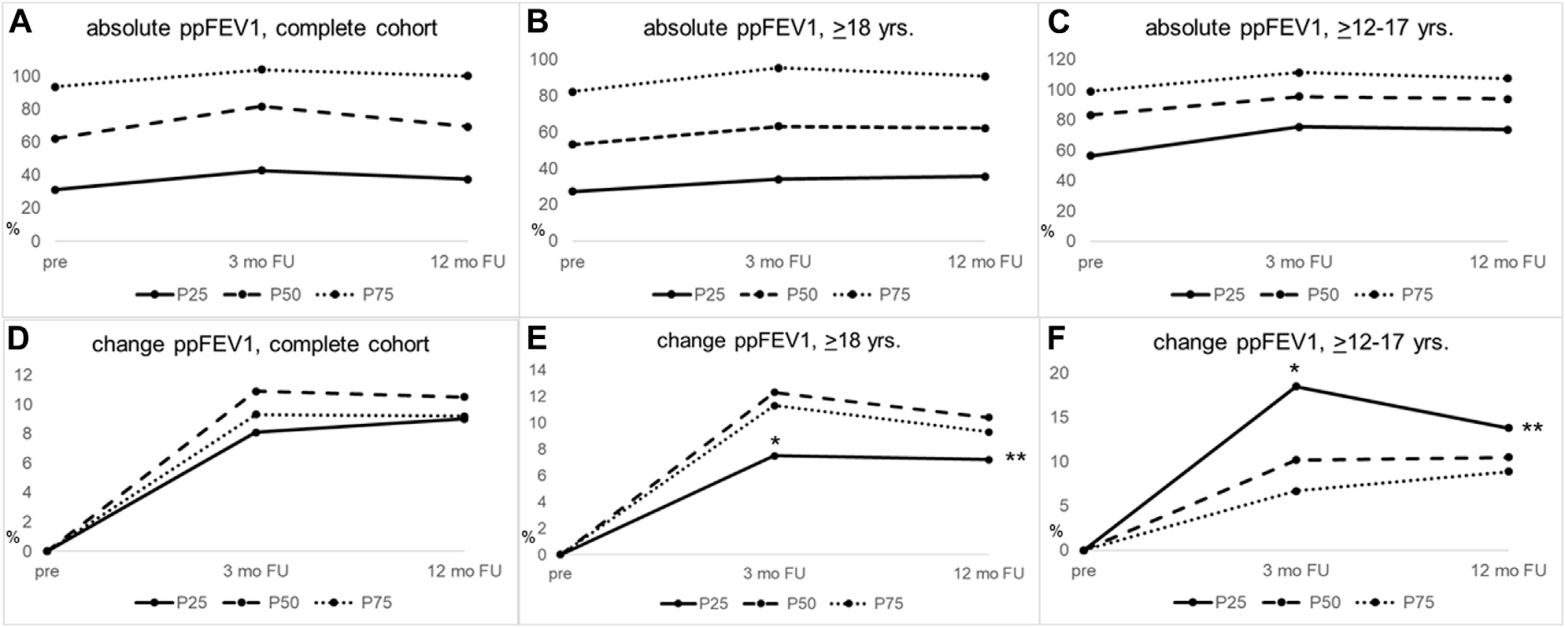
PpFEV1 gains in response to ETI therapy differ between groups of lung disease severity within the same age stratum and are significantly larger in adolescents with severe lung disease, compared to adults with severe lung disease. **(A–C)** Median absolute values of ppFEV1 for **(A)** the complete cohort, **(B)** pwCF ≥18 years, and **(C)** pwCF ≥12–17 years of age. **(D–F)** Change from baseline ppFEV for **(D)** the complete cohort, **(E)** pwCF ≥18 years of age, and **(F)** pwCF ≥12–17 years. Adults (middle column) with less severe lung disease (≥P75) showed significantly higher ppFEV1 increase than pwCF with worse lung disease (≤P25) 3 months after ETI initiation, *p*-value = 0.019. Adolescents (right column) with worse lung disease (≤P25) had significantly higher ppFEV1 gains than those with less severe lung disease (P50) 3 months after ETI initiation, *p*-value = 0.027, whereas in adults, the group with lower lung function at ETI initiation (≤P25) showed less significant ppFEV1 gains at 3 months than the group with the highest ppFEV1 percentile (≤P75; *p* = 0.025). PpFEV1 gains at 3 and 12 months of ETI treatment were significantly higher in adolescents with worse lung disease (≤P25) than adults (≤P25) at 3 (^*^ *p* = 0.002) and 12 months (^**^ *p* = 0.012). Severity of lung disease was calculated, as outlined in materials and methods. FU, follow-up; mo, months; ≤P25, baseline ppFEV1 ≤25th age-specific percentile; P50, baseline ppFEV1 26th–74th age-specific percentile; ≥P75, baseline ppFEV1 ≥75th age-specific percentile; yrs, years.

As expected, absolute BMI values at baseline were significantly lower in the participants with the lowest lung function [19.1 (16.8–21.4) vs. 19.3 (17.0–21.2) vs. 20.2 (18.0–22.2), *p* = 0.041, for ppFEV1 ≤P25 vs. P50 vs. ≥P75, respectively]. BMI changes 3 and 12 months after initiation of ETI did show significant group differences (*p* = 0.001 at 3 months; *p* = 0.022 at 12 months), suggesting higher BMI gains in the participants with lower lung function (+1.2 (0.6–2.1) vs.+ 0.7 (0.0–1.4) vs. + 0.7 (0.0–1.3) for ppFEV1 ≤P25 vs. P50 vs. ≥P75, respectively, at 3-month FU and +2.2 (0.6–3.5) vs. +1.1 (0.3–2.6) vs. +1.1 (0.4–2.3) for ppFEV1 ≤P25 vs. P50 vs. ≥P75, respectively, at 12-month FU (Supplementary Table S1; Figure 1, left column).

### Lung disease severity is associated with larger ppFEV1 improvements in adolescents and children but not adults

When stratifying for the severity of lung disease in the adolescents (≥12–17 years, Table 2), we observed significantly higher improvements in ppFEV1 in participants with the worst baseline lung function at 3 months post-ETI initiation (ppFEV1 +18.5 (10.6–28.2) vs. +10.2 (5.2–17.7) vs. +6.7 (2.3–14.5), *p* = 0.027 for ppFEV1 ≤P25 vs. P50 vs. ≥P75, respectively). This difference abated at 12 months post-ETI initiation (+13.8 (11.3–24.3) vs. +10.5 (5.6–24.0) vs. +8.9 (2.9–11.9), *p* = 0.09 for ppFEV1 ≤P25 vs. P50 vs. ≥P75, respectively) (Table 2; Figure 1, right column).

**TABLE 2:** Patients characteristics of the total cohort stratified for age-dependent severity of lung disease as outlined in materials and methods. PpFEV1 gains and BMI gains distribute unevenly between the age-specific ppFEV1 groups and also between the age groups when comparing groups of similar age-specific lung function severity. *p*-values refer to Kruskal-Wallis or Mann-Whitney-U inter-group comparisons for continuous variables and chi-squared tests for discontinuous variables. ^**f**^ *p*-value = 0.027 ≤P25 vs. P50 group; ^**g**^ *p*-value = 0.019 ≤P25 vs. ≥P75 groups; ^**h**^ *p*-value = 0.036 ≤P25 vs. ≥P75; ^**i**^ *p*-value = 0.006 ≤P25 vs. P50 group; ^**j**^ *p*-value = 0.026 P50 vs. ≥P75 group; ^**k**^ *p*-value = 0.066 ≤P25 vs. P50 group^; **l**^ *p*-value = 0.046 ≤P25 vs. *≥*P75; ^**m**^ *p* = 0.021; ^*^ *p*-value = 0.002 for ppFEV1 gains at three months of ETI therapy between ≥12–17 years age group vs. ≥18 years age group, ^**^ *p*-value = 0.012 for ppFEV1 gains at 12 months of ETI therapy between ≥12–17 years age group vs. ≥18 years age group. BMI, Body-mass-index; ETI, Elexacaftor/tezacaftor/ivacaftor; FU, Follow up; IQR, Inter quartile range; mo., month; yrs, years; ≤P25, baseline ppFEV1 ≤25^th^ age-specific percentile; P50, baseline ppFEV1 26^th^–74^th^ age-specific percentile; ≥P75, baseline ppFEV1 ≥75^th^ age-specific percentile.

≥12–17 years (*n* = 41)				
Characteristics	≤P25 ppFEV1 (*n* = 10)	P50 ppFEV1 (*n* = 21)	≥P75 ppFEV1 (*n* = 10)	*p*-value
Age at start (yrs), median (IQR)	15.2 (12.7−17.0)	13.2 (12.2−15.4)	13.8 (13.7−15.8)	0.190
Sex (female, %)	70.0	61.9	70.0	0.861
CFTR_prior ETI (%)	40.0	14.3	10.0	0.164
F508del homozygous (%)	60.0	14.3	50.0	0.021
FEV1% at start, median (IQR)	56.4 (50.2−61.9) (*n* = 10)	83.4 (73.9−91.3) (*n* = 21)	98.9 (95.5−110.9) (*n* = 10)	—
FEV1% at FU 3 mo, median (IQR)	75.6 (70.6−82.2) (*n* = 10)	95.6 (82.8−104.9) (*n* = 21)	111.4 (100.2−118.4)(*n* = 10)	—
FEV1% at FU 12 mo, median (IQR)	73.6 (64.4−81.7) (*n* = 10)	94.0 (86.6−107.4) (*n* = 19)	107.6 (104.4−120.4)(*n* = 10)	—
∆ FEV1% _(FU 3 mo.–start)_, median (IQR)	18.5 (10.6−28.2)^**f**,^ * (*n* = 10)	10.2 (5.2−17.7)^**f**^ (*n* = 21)	6.7 (2.3−14.5) (*n* = 10)	0.027
∆ FEV1% _(FU 12 mo.–start)_, median (IQR)	13.8 (11.3−24.3)** (*n* = 10)	10.5 (5.6−24.0) (*n* = 19)	8.9 (2.9−11.9) (*n* = 10)	0.090
BMI (kg/m^2^) at start, median (IQR)	17.8 (16.1−20.9) (*n* = 10)	18.1 (16.8−20.3) (*n* = 21)	19.4 (17.8−20.4) (*n* = 10)	0.495
BMI (kg/m^2^) at FU 3 mo, median (IQR)	19.7 (17.8−23.7) (*n* = 10)	18.9 (17.2−21.4) (*n* = 21)	21.1 (17.7−22.5) (*n* = 10)	0.437
BMI (kg/m^2^) at FU 12 mo, median (IQR)	19.5 (18.3−24.9) (*n* = 10)	19.1 (17.8−20.8) (*n* = 19)	21.1 (18.8−22.0) (*n* = 10)	0.221
∆ BMI (kg/m^2^) _(FU 3 mo.–start)_, median(IQR)	2.0 (1.4−2.4)^**g**^ (*n* = 10)	1.0 (0.3−1.5) (*n* = 21)	0.6 (−0.0−2.3)^**g**^ (*n* = 10)	0.034
∆ BMI (kg/m^2^) _(FU 12 mo.–start)_,median(IQR)	2.6 (−0.1−4.5) (*n* = 10)	1.1 (0.1−2.4) (*n* = 19)	1.1 (0.6−2.6) (*n* = 10)	0.323

≥18 years (*n* = 143)				
Characteristics	≤P25 ppFEV1 (*n* = 35)	P50 ppFEV1 (*n* = 73)	≥P75 ppFEV1 (*n* = 35)	*p*-value
Age at start (yrs), median (IQR)	35.2 (23.4−41.6)	28.0 (23.3−33.8)	24.5 (20.3−32.9)	0.092
Gender (female, %)	48.6	49.3	48.6	0.996
CFTR_prior ETI (%)	48.6	54.8	42.9	0.495
F508del homozygous (%)	65.7	58.9	57.1	0.729
FEV1% at start, median (IQR)	27.2 (23.4−30.4) (*n* = 35)	53.1 (45.4−60.7) (*n* = 73)	82.3 (77.0−92.9) (*n* = 35)	—
FEV1% at FU 3 mo, median (IQR)	34.1 (28.1−39.9) (*n* = 35)	63.3 (55.6−82.5) (*n* = 73)	95.6 (86.9−107.3) (*n* = 35)	—
FEV1% at FU 12 mo, median (IQR)	35.5 (28.4−41.3) (*n* = 35)	62.2 (55.2−78.1) (*n* = 73)	90.9 (84.4−106.3) (*n* = 35)	—
∆ FEV1% _(FU 3 mo.–start)_, median (IQR)	7.5 (3.7−10.7)^**h**,^ * (*n* = 35)	12.3 (6.2−21.6) (*n* = 73)	11.3 (6.0−16.6)^**h**^ (*n* = 35)	0.025
∆ FEV1% _(FU 12 mo.–start)_, median (IQR)	7.2 (3.5−11.8)** (*n* = 35)	10.4 (5.6−22.0) (*n* = 73)	9.3 (6.2−14.2) (*n* = 35)	0.121
BMI (kg/m^2^) at start, median (IQR)	20.1 (17.6−21.9)^**i**^ (*n* = 35)	20.5 (18.6−22.3)^**i**, **j**^ (*n* = 73)	21.7 (20.2−24.3)^**j**^ (*n* = 35)	0.005
BMI (kg/m^2^) at FU 3 mo, median (IQR)	20.9 (19.3−24.1) (*n* = 35)	21.3 (19.8−23.5) (*n* = 73)	22.3 (21.2−25.7) (*n* = 35)	0.089
BMI (kg/m^2^) at FU 12 mo, median (IQR)	22.2 (19.5−24.9) (*n* = 35)	22.0 (20.1−24.2) (*n* = 73)	22.4 (20.8−26.5) (*n* = 35)	0.438
∆ BMI (kg/m^2^) _(FU 3 mo.–start)_, median(IQR)	1.2 (0.6−2.2)^**k**^ (*n* = 35)	0.9 (0.3−1.6)^**k**^ (*n* = 73)	0.7 (0.0−1.5) (*n* = 35)	0.065
∆ BMI (kg/m^2^) _(FU 12 mo.–start)_,median(IQR)	2.0 (0.8−3.4) (*n* = 35)	1.1 (0.3−2.7) (*n* = 73)	1.1 (0.0−2.2) (*n* = 35)	0.048
≥6–11 years (*n* = 42)				
Characteristics	≤P25 ppFEV1 (*n* = 10)	P50 ppFEV1 (*n* = 22)	≥P75 ppFEV1 (*n* = 10)	*p*-value
Age at start (yrs), median (IQR)	10.2 (7.1−11.3)	9.0 (7.3−10.5)	8.1 (6.7−9.3)	0.203
Sex (female, %)	50.0	45.5	50.0	0.958
CFTR_prior ETI (%)	60.0	50.0	50.0	0.858
F508del homozygous (%)	60.0	50.0	60.0	0.809
FEV1% at start, median (IQR)	70.7 (61.4−80.7) (*n* = 10)	87.7 (82.9−95.0) (*n* = 22)	102.1 (100.6−105.4)(*n* = 10)	—
FEV1% at FU 3 mo, median (IQR)	83.7 (74.1−94.5) (*n* = 10)	98.8 (88.7−101.9) (*n* = 22)	107.5 (105.1−112.7)(*n* = 10)	—
∆ FEV1% _(FU 3 mo.–start)_, median (IQR)	19.8 (11.6−28.3) (*n* = 10)	8.4 (5.0−13.3) (*n* = 22)	5.2 (1.3−7.8) (*n* = 10)	0.126
BMI (kg/m^2^) at start, median (IQR)	16.4 (15.0−18.1) (*n* = 10)	15.3 (14.4−15.8) (*n* = 22)	16.1 (15.3−16.9) (*n* = 10)	0.039
BMI (kg/m^2^) at FU 3 mo, median (IQR)	17.0 (14.9−19.3)^**l**^ (*n* = 10)	15.1 (14.3−15.8) (*n* = 22)	16.2 (15.3−17.1)^**l**^ (*n* = 10)	0.039
∆ BMI (kg/m^2^) _(FU 3 mo.–start)_, median(IQR)	0.8 (0.2−1.3)^**m**^ (*n* = 10)	0.2 (−0.4−0.3) (*n* = 22)	0.3 (−0.1−0.8)^**m**^ (*n* = 10)	0.026

The more pronounced ppFEV1 gains in the adolescent participants with worse lung function were in contrast to the adult patients. Here, lower lung function at baseline was associated with significantly fewer improvements at 3 months post-ETI initiation (+7.5 (3.7–10.7) vs. +12.3 (6.2–21.6) vs. +11.3 (6.0–16.6), *p* = 0.025 for ppFEV1 ≤P25 vs. P50 vs. ≥P75, respectively), which abated at 12 months (7.2 (3.5–11.8) vs. 10.4 (5.6–22.0) vs. 9.3 (6.2–14.2), *p* = 0.121 for ppFEV1 ≤P25 vs. P50 vs. ≥P75, respectively) (Table 2; Figure 1 middle column).

When comparing adolescents (≥12–17 years) and adults with worse lung function (≤P25) for ppFEV1 gain after 3 and 12 months of ETI initiation, adolescents showed significantly higher improvements at both time points than adults (*p* = 0.002 and *p* = 0.012 for 3 and 12 months, respectively, Table 2).

Complementing our cohort with data from patients between 6 and 11 years of age supported the finding that in younger patients, those with more severe lung disease show higher gains in ppFEV1 3 months after ETI initiation (Table 2). At baseline, as expected, this cohort showed an even more preserved lung function in the three ppFEV1 percentile groups than adults and adolescents [ppFEV1 baseline: 70.7 (61.4–80.7) vs. 87.7 (82.9–95.0) vs. 102.1 (100.6–105.4) for ppFEV1 ≤P25 vs. P50 vs. ≥P75, respectively]. In these younger children, the previously described effect of a more pronounced ppFEV1 gain in adolescent patients with a lower lung function was also seen. However, this change did not reach statistical significance [+19.8 (0.4–26.0) vs. +8.4 (5.0–13.3) vs. +5.2 (1.3–7.8), *p* = 0.126 for ≤P25 vs. P50 vs. ≥P75, respectively], (Table 2; Supplementary Table S1A). Furthermore, these higher ppFEV1 gains in those children within the worst ppFEV1 stratum were not significantly different from those of the adolescents or the adult group. Yet, when comparing all children ≥6–17 yrs with a worse lung function (*n* = 20 ≤P25) than adults with ppFEV1 ≤P25, this finding attained significance with a higher gain of ppFEV1 in children (children and adolescents: +19.3 (8.0–27.3) vs. adults: +7.5 (3.7–10.7), *p* = 0.007) 3 months after ETI initiation (data not shown).

### More severe lung disease is associated with larger BMI improvement regardless of age

**FIGURE 2:**
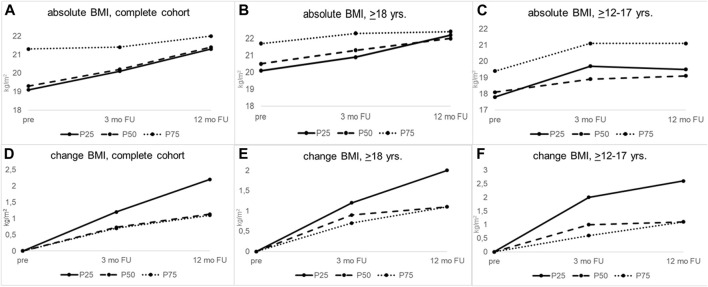
BMI gains in response to ETI therapy differ between groups of lung disease severity within the same age stratum but do not show differences in response between adolescents and adults. **(A–C)** Median absolute values of BMI for **(A)** the complete cohort, **(B)** pwCF ≥18 years, and **(C)** pwCF ≥12–17 years of age. **(D–F)** Change from baseline BMI for **(D)** the complete cohort, **(E)** pwCF ≥18 years of age, and **(F)** pwCF ≥12–17 years. In the overall cohort (left column), patients with worse lung function (≤P25) had significantly higher BMI gains at 3 (*p* = 0.001) and 12 months (*p* = 0.022) after ETI initiation than those with medium or good lung function (P50; ≥P75). The same effect was confirmed in the other age groups. Inter-age comparison did not show any statistical differences for BMI gains (middle and right column). FU, follow-up; mo, months; ≤P25, baseline ppFEV1 ≤25th age-specific percentile; P50, baseline ppFEV1 26th–74th age-specific percentile; ≥P75, baseline ppFEV1 ≥75th age-specific percentile; yrs, years.

In the adolescent participants, there was no statistical difference in the absolute baseline BMI values when stratified according to the severity of lung disease prior to ETI initiation (Table 2; Figure 2, right column). Absolute BMI values at 3 or 12 months post-ETI initiation also did not differ statistically according to the severity of lung disease in this age group (Table 2). Participants with more severe lung disease showed higher BMI gains than those with a better lung function at 3 (≤P25 = 2.0 vs. P50 = 1.0 vs. ≥P75 = 0.6 kg/m*2; *p* = 0.034) and 12 months (≤P25 = 2.6 vs. P50 = 1.1 vs. ≥P75 = 1.1 kg/m*2; *p* = 0.323), although these differences were statistically significant only at 3 months post-ETI initiation (Table 2; Figure 2, right column).

In contrast to the adolescent participants, adults with the most severe lung function had significantly worse absolute BMI values at baseline than patients with a better lung function (20.1 (17.6–21.9) vs. 20.5 (18.6–22.3) vs. 21.7 (20.2–24.3), *p* = 0.005 for ppFEV1 ≤P25 vs. P50 vs. ≥P75 respectively) (Table 2). These differences were lost 3 and 12 months after initiation of ETI (*p* = 0.089 and *p* = 0.438), suggesting more significant improvements in response to ETI in those participants with a worse lung function. Indeed, for adults, significantly larger improvements in BMI were observed in those pwCF with the worst lung function after both 3 (≤P25 = +1.2 vs. P50 = 0.9 vs. ≥P75 = 0.7 kg/m*2; *p* = 0.065) and 12 months (≤P25 = 2.0 vs. P50 = 1.1 vs. ≥P75 = 1.1; *p* = 0.048) (Table 2).

Interestingly, in pwCF ≥6–11 years of age (Table 2; Supplementary Table S1B), BMI improvements at 3 months were also larger in those with more severe lung function impairment (+0.8 (0.2–1.3) vs. +0.2 (−0.4-0.3) vs. +0.3 (−0.1-0.8), *p* = 0.026 for ppFEV1 ≤P25 vs. P50 vs. ≥P75, respectively).

## Discussion

Our analyses provide initial data, which suggest that ETI response, as reflected in ppFEV1 and BMI levels, is dependent on age at initiation and lung disease severity. Specifically, the data show that children and adolescents with more severe lung disease (defined as age-group-specific ≤25% percentile ppFEV1) exhibited higher ppFEV1 gains at 3 and 12 months after ETI initiation than those with a better lung function at baseline in the same age group. Furthermore, inter-age comparisons of those participants affected most severely with respect to lung disease (age-specific ppFEV1 ≤P25) showed higher ppFEV1 gains in children and adolescents than adults at 3 and 12 months after ETI introduction. These findings are supported by regression analyses in the sub-group of all pwCF affected most severely (ppFEV1 ≤P25 at ETI initiation), which identified an association of younger age with higher ppFEV1 gains at 3 and at 12 months after initiation of ETI therapy.

To the best of our knowledge, our data are the first to identify larger lung function improvements toward CFTR modulator therapy in adolescents (Table 2; Figure 1), and adolescents and children (data shown in the previous section) with more severe lung function impairment than adults with more severe lung function impairment.

Previous studies also indicated a more favorable response profile of children compared to adults with regard to ppFEV1. Earlier introduction of IVA or LUM/IVA led to better preservation of lung function, a larger impact on PEx (Merlo et al., 2022), and a higher ppFEV1 response (Bui et al., 2021). More recently, Muilwijk et al., in a long-term study on data from the Dutch CF registry on real-world data on LUM/IVA responses, also found larger increases of ppFEV1 in pwCF with more advanced lung disease (Muilwijk et al., 2022). Their study incorporated longer follow-up data and more participants than most previous analyses on LUM/IVA, possibly unveiling effects not previously identifiable due to limited numbers or study duration. It seems conceivable that the larger functional improvements achieved by ETI compared to LUM/IVA (Graeber et al., 2021; Graeber et al., 2022a; Graeber et al., 2022b) provide the necessary statistical power to this study of ETI despite the lower numbers of participants. Our regression analyses support age as a central factor for ppFEV1 responses as they showed effects of age at ETI initiation on ppFEV1 gains both at 3- and 12-month follow-ups for both the entire cohort and those with most severe lung function impairment. Together, Muilwijk’s and our findings suggest that younger patients with more severe lung disease experience higher gains in ppFEV1 in response to CFTR modulation.

Our analyses on improvements of ppFEV1 in those pwCF with severe lung disease are in a similar range as previous data by Burgel et al. (2021) and Carnovale et al. (2022) and do not refute their conclusions that the pwCF with the most advanced lung disease do benefit considerably from ETI. They rather extend their observation to younger age groups. Furthermore, our data indicate that in adult pwCF, compared to adolescent pwCF, those with more severe lung disease at ETI initiation take longer time to improve as the differences in ppFEV1 gains in adult pwCF, which were significant at 3 months between the three groups of lung disease severity, abated at 12 months (Table 2).

Unlike Muilwijk et al., our data failed to identify that adolescents or children show a more favorable BMI trajectory than adults (Muilwijk et al., 2022). Our data did unveil stronger BMI increases in those individuals with more severe lung function. However, these effects were seen in all age groups. These findings were supported by our regression analyses, which showed a significant impact of ppFEV1 at initiation for BMI gains at 3 months but not after 12 months of ETI therapy, whereas age only affected BMI gains after 12 months but not after 3 months, indicating a complex interplay of age and severity of lung disease at ETI initiation, for which at present, we fail to identify uni-directional effects.

Our analyses failed to identify the effects of gender on absolute values of ppFEV1 or BMI or ppFEV1 or BMI gains attained through ETI therapy, where female subjects have been shown to respond more favorably toward IVA with regard to annualized pulmonary exacerbation (PEx) rates and respond to LUM/IVA with larger BMI gains (Secunda et al., 2020; Muilwijk et al., 2021). Yet, the inclusion of multi-centric data, some of which stemming from more controlled clinical trials, makes a direct comparison difficult. These studies also addressed other CFTR modulators and thus might also suggest that ETI lacks gender-dependent response profiles seen with other CFTR modulators.

Interestingly, the models we built also failed to indicate an effect of prior CFTR modulator therapy on absolute ppFEV1 or BMI, or ppFEV1 or BMI gains. The PROMISE Study Group showed that the highest average changes in ppFEV1 were in those pwCF previously using no modulator or a two-drug combination (Nichols et al., 2022). Our findings on superior ppFEV1 gains in adolescents with severe lung function impairment compared to adults within the same range of lung function impairment might recapitulate the observations made in PROMISE since the adolescent age group contains a significantly lower proportion of modulator therapy prior to ETI initiation (19.5%) vs. the adult group (50.3%) (*p* = 0.001, Table 1). However, modulator use in children 6–11 years of age was comparable between this age group and adults, and nonetheless, the children with severe lung function impairment showed higher ppFEV1 gains than the adults with severe lung function impairment.

Our analysis has important limitations, particularly due to the heterogeneity of our subpopulations. In that line, our study cohorts differ significantly in the proportion of previous CFTR modulator therapy prior to ETI initiation (adolescents 19.5% vs. adults 50% vs. children 6–11 years 52%). Our smaller cohort with limited follow-up data may be underpowered to produce conclusive results, particularly due to unaccounted effects of previous CFTR modulator therapy. On one hand, responses to CFTR modulator therapy may be more favorable in pwCF who had had prior therapy compared to those without because the latter group may have developed irreversible changes in the intervening time which preclude further positive responses. On the other hand, improvements in response to prior CFTR modulator therapy might eventually provide a “ceiling” effect where further improvements in either ppFEV1 or BMI cannot be observed anymore. Yet, children and adolescents, with the worst lung function (≤P25) comprised the highest proportion of previous CFTR modulator therapy (adolescents 40.0%; children 60.0%) in a comparable range to adults (48.6%). Regression analysis in this cohort with regard to the impact of previous modulator therapy did reveal significant influences of this variable.

In addition, our age sub-groups differ in the proportion of deltaF508 homozygosity, which might impact the disease severity. Interestingly, our focus sub-group (spirometry ≤P25) contains a comparable proportion of deltaF508 homozygosity across all age groups (60%–66%), which improves the comparability of these subpopulations. Along this line, other publications also failed to identify the effects of genotype–phenotype correlations, albeit also at small cohort sizes (Carnovale et al., 2022). Still, given our cohort size and its mono-centricity, we cannot exclude that previous CFTR modulator therapy or genotype influences response to ETI. Larger cohorts, preferably registry-based data and/or longer observation periods, are necessary to disentangle these aspects.

Clinical data concerning longitudinal evolvement of pulmonary function and anthropometry, supporting age-dependent effects, are yet lacking for ETI. Such data are of particular importance in view of the large number of young patients, which became and will still become eligible for ETI. Due to previous diagnostic and therapeutic improvements, this patient population will initiate CFTR modulator therapy in an unprecedented state of health. This, combined with their young age, will render the monitoring of clinical changes in response to CFTR modulator therapy, particularly challenging. Data, as we present here, which support an association of the magnitude of response toward ETI and younger age, might lend credibility to a comprehensive approach toward the initiation of ETI at the earliest age possible, as well as advocating treatment continuation even in cases, where the subjective or objective benefits might not be immediately observable due to low functional impairment at ETI initiation.

In light of the first study demonstrating long-term lung function stability in those individuals treated with ETI (Lee et al., 2022), the earliest possible initiation of ETI promises to add an unprecedented therapeutic value for pwCF. Our study is one of the few studies, which incorporates longitudinal data from pwCF aged 6+ years. Our data partially confirm our hypothesis that early initiation of ETI will prove most beneficial but limit this confirmation to those with most severe lung disease. Furthermore, our study design limits its transfer to other cohorts and clinical settings. Due to licensing timing, we included a much larger group of adults in our study than we were able to include adolescents or children, the latter of which we could only analyze after 3 months but not yet after 12 months of ETI therapy. In addition, our severity and age-specific sub-groups are of even lower numbers. These limitations may lead to statistical bias; therefore, results need to be interpreted cautiously. Our retrospective approach is by design exploratory; therefore, results need to be cautiously interpreted. We cannot exclude that follow-up data on larger groups of pwCF with longer follow-up trajectories might unveil some effects we failed to identify. In that same line, some of the identified effects might no longer be identifiable in a more heterogeneous population. Our data demonstrate a wide age range and disease severity but stem from a single CF center in Germany, albeit one of Germany’s larger centers. Although this mono-centric approach permits analysis of pwCF treated according to similar standards across all ages, this approach necessarily limits generalizability, which can only be gained from larger, preferably prospective clinical trials or register-based studies. We, thus, advocate patience until larger and longer studies validate our findings by interrogating the drivers of ETI response, including, in particular, factors such as gender, age, and clinical status at initiation.

## Data Availability

The raw data supporting the conclusion of this article will be made available by the authors, without undue reservation.

## Supplementary material

SUPPLEMENTARY FIGURE S1 Gains in ppFEV1 are highest in the groups with the most severe pre-existing lung disease. **(A)** ppFEV1 gains and **(B)** BMI gains from baseline to 3 months of ETI therapy, stratified for the severity of lung disease for the age groups ≥12–17 yrs and ≥6–11 years. In both age groups, highest gains of ppFEV1 were seen in children with worst lung disease (≤P25). Highest gains in BMI were seen in adolescents within the group with the most severe lung disease (≤P25). FU, follow-up; mo, months; ≤P25, baseline ppFEV1 ≤25th age-specific percentile; P50, baseline ppFEV1 26th–74th age-specific percentile; ≥P75, baseline ppFEV1 ≥75th age-specific percentile; yrs, years.

## References

[B1] Aalbers B. L. Hofland R. W. Bronsveld I. de Winter-de Groot K. M. Arets H. G. M. de Kiviet A. C. (2021). Females with cystic fibrosis have a larger decrease in sweat chloride in response to lumacaftor/ivacaftor compared to males. J. Cyst. Fibros. 20, e7–e11. 10.1016/j.jcf.2020.05.004 32448708

[B2] Bui S. Masson A. Enaud R. Roditis L. Dournes G. Galode F. (2021). Long-term outcomes in real life of lumacaftor-ivacaftor treatment in adolescents with cystic fibrosis. Front. Pediatr. 9, 744705. 10.3389/fped.2021.744705 34869102 PMC8634876

[B3] Burgel P. R. Durieu I. Chiron R. Ramel S. Danner-Boucher I. Prevotat A. (2021). Rapid improvement after starting elexacaftor-tezacaftor-ivacaftor in patients with cystic fibrosis and advanced pulmonary disease. Am. J. Respir. Crit. Care Med. 204, 64–73. 10.1164/rccm.202011-4153OC 33600738

[B4] Carnovale V. Iacotucci P. Terlizzi V. Colangelo C. Ferrillo L. Pepe A. (2022). Elexacaftor/Tezacaftor/Ivacaftor in Patients with Cystic Fibrosis Homozygous for the F508del Mutation and Advanced Lung Disease: A 48-Week Observational Study, J. Clin. Med. 11, 1021. 10.3390/jcm11041021 35207295 PMC8876133

[B5] Davies J. C. Cunningham S. Harris W. T. Lapey A. Regelmann W. E. Sawicki G. S. (2016). Safety, pharmacokinetics, and pharmacodynamics of ivacaftor in patients aged 2–5 years with cystic fibrosis and a CFTR gating mutation (KIWI): An open-label, single-arm study. Lancet Respir. Med. 4, 107–115. 10.1016/S2213-2600(15)00545-7 26803277 PMC6734927

[B6] Davies J. C. Wainwright C. E. Sawicki G. S. Higgins M. N. Campbell N. Harris C. (2021). Ivacaftor in infants aged 4 to <12 months with cystic fibrosis and a gating mutation. Results of a two-part phase 3 clinical trial. Am. J. Respir. Crit. Care Med. 203, 585–593. 10.1164/rccm.202008-3177OC 33023304 PMC7924576

[B7] Dittrich A-M. Chuang S. Y. (2022). Dual CFTR modulator therapy efficacy in the real world: Lessons for the future. ERJ Open Res. 8, 00464–02022. 10.1183/23120541.00464-2022 36382239 PMC9661234

[B8] Durmowicz A. G. Witzmann K. A. Rosebraugh C. J. Chowdhury B. A. (2013). Change in sweat chloride as a clinical end point in cystic fibrosis clinical trials: The ivacaftor experience. Chest 143 (1), 14–18. 10.1378/chest.12-1430 23276841

[B9] Graeber S. Y. Boutin S. Wielpütz M. O. Joachim C. Frey D. L. Wege S. (2021). Effects of lumacaftor–ivacaftor on lung clearance index, magnetic resonance imaging, and airway microbiome in Phe508del homozygous patients with cystic fibrosis. Ann. Am. Thorac. Soc. 18, 971–980. 10.1513/AnnalsATS.202008-1054OC 33600745

[B10] Graeber S. Y. Renz D. M. Stahl M. Pallenberg S. T. Sommerburg O. Naehrlich L. (2022a). Effects of elexacaftor/tezacaftor/ivacaftor therapy on lung clearance index and magnetic resonance imaging in patients with cystic fibrosis and one or two *F508del* alleles. Am. J. Respir. Crit. Care Med. 206, 311–320. 10.1164/rccm.202201-0219oc 35536314

[B11] Graeber S. Y. Vitzthum C. Pallenberg S. T. Naehrlich L. Stahl M. Rohrbach A. (2022b). Effects of elexacaftor/tezacaftor/ivacaftor therapy on CFTR function in patients with cystic fibrosis and one or two F508del alleles. Am. J. Respir. Crit. Care Med. 205, 540–549. 10.1164/rccm.202110-2249OC 34936849

[B12] Heijerman H. G. M. McKone E. F. Downey D. G. Braeckel E. V. Rowe S. M. Tullis E. (2019). Efficacy and safety of the elexacaftor plus tezacaftor plus ivacaftor combination regimen in people with cystic fibrosis homozygous for the F508del mutation: A double-blind, randomised, phase 3 trial. Lancet 394, 1940–1948. 10.1016/S0140-6736(19)32597-8 31679946 PMC7571408

[B13] Lee T. Sawicki G. S. Altenburg J. Millar S. J. Geiger J. M. Jennings M. T. (2022). Effect of Elexacaftor/Tezacaftor/Ivacaftor on annual rate of lung function decline in people with cystic fibrosis. J. Cyst. Fibros. 2022, S1569–S1993. 10.1016/j.jcf.2022.12.009 36581485

[B14] McNamara J. J. McColley S. A. Marigowda G. Liu F. Tian S. Owen C.-A. (2019). Safety, pharmacokinetics, and pharmacodynamics of lumacaftor and ivacaftor combination therapy in children aged 2–5 years with cystic fibrosis homozygous for F508del-CFTR: An open-label phase 3 study. Lancet Respir. Med. 7, 325–335. 10.1016/S2213-2600(18)30460-0 30686767

[B15] Merlo C. McGarry L. Thorat T. Nguyen C. DerSarkissian M. Muthukumar A. (2022). WS17.03 initiating ivacaftor (IVA) at younger vs older ages improves pulmonary outcomes in people with cystic fibrosis (pwCF): A long-term real-world study. J. Cyst. Fibros. 21, S33–S34. 10.1016/S1569-1993(22)00251-X

[B16] Mesinele J. Ruffin M. Guillot L. Boelle P. Y. Corvol H. (2022). WS07.02 lumacaftor/ivacaftor in people with cystic fibrosis: Factors predisposing the response and impact on lung function decline. J. Cyst. Fibros. 21, S13. 10.1016/S1569-1993(22)00190-4 34629287

[B17] Middleton P. G. Mall M. A. Drevinek P. Lands L. C. McKone E. F. Polineni D. (2019). Elexacaftor-Tezacaftor-Ivacaftor for cystic fibrosis with a single Phe508del allele. N. Engl. J. Med. 381, 1809–1819. 10.1056/NEJMoa1908639 31697873 PMC7282384

[B18] Muilwijk D. Bierlaagh M. van Mourik P. Kraaijkamp J. van der Meer R. Heijerman H. (2021). Prediction of real-world long-term outcomes of people with CF homozygous for the F508del mutation treated with CFTR modulators. J. Pers. Med. 11, 1376. 10.3390/jpm11121376 34945848 PMC8707616

[B19] Muilwijk D. Zomer-van Ommen D. D. Gulmans V. A. M. Eijkemans M. J. van der Ent C. K. Altenburg J. (2022). Long-term effectiveness of dual CFTR modulator treatment of cystic fibrosis. ERJ Open Res. 8, 00204–02022. 10.1183/23120541.00204-2022 36382237 PMC9661249

[B20] Nichols D. P. Paynter A. C. Heltshe S. L. Donaldson S. H. Frederick C. A. Freedman S. D. (2022). Clinical effectiveness of elexacaftor/tezacaftor/ivacaftor in people with cystic fibrosis: A clinical trial. Am. J. Respir. Crit. Care Med. 205, 529–539. 10.1164/rccm.202108-1986OC 34784492 PMC8906485

[B21] Quanjer P. H. Stanojevic S. Cole T. J. Baur X. Hall G. L. Culver B. H. (2012). Multi-ethnic reference values for spirometry for the 3–95-yr age range: The global lung function 2012 equations. Eur. Respir. J. 40, 1324–1343. 10.1183/09031936.00080312 22743675 PMC3786581

[B22] Rosenfeld M. Wainwright C. E. Higgins M. Wang L. T. McKee C. Campbell D. (2018). Ivacaftor treatment of cystic fibrosis in children aged 12 to <24months and with a CFTR gating mutation (ARRIVAL): A phase 3 single-arm study. Lancet Respir. Med. 6, 545–553. 10.1016/S2213-2600(18)30202-9 29886024 PMC6626762

[B23] Secunda K. E. Guimbellot J. S. Jovanovic B. Heltshe S. L. Sagel S. D. Rowe S. M. (2020). Females with cystic fibrosis demonstrate a differential response profile to ivacaftor compared with males. Am. J. Respir. Crit. Care Med. 201 (8), 996–998. 10.1164/rccm.201909-1845LE 31841644 PMC7159427

[B24] Zemanick E. T. Taylor-Cousar J. L. Davies J. Gibson R. L. Mall M. A. McKone E. F. (2021). A Phase 3 Open-Label Study of Elexacaftor/Tezacaftor/Ivacaftor in Children 6 through 11 Years of Age with Cystic Fibrosis and at Least One *F508del* Allele. Am. J. Respir. Crit. Care Med. 203, 1522–1532. 10.1164/rccm.202102-0509OC 33734030 PMC8483230

