# Supplementary material for: Spirometric and anthropometric improvements in response to elexacaftor/tezacaftor/ivacaftor depending on age and lung disease severity
Source: Front Pharmacol. 2023 Jul 4;14:1171544. doi: 10.3389/fphar.2023.1171544 (PMC10352657; doi:10.3389/fphar.2023.1171544)
Supplement: Supplementary file 1 [file Table1.DOCX]

**Supplemental Table 1: Patients characteristics of the total cohort stratified for severity of lung disease.** Stratification for severity of lung diseae, revealed significantly higher BMI values at baseline for individuals with less severe lung disease (19.1 vs. 19.3 vs. 20.2 kg/m^2^, p=0.0041) as well as significantly higher BMI gains at three and 12 months after ETI initiation (1.2 vs. 0.7. vs. 0.7 kg/m^2^, p=0.001 and 2.2 vs 1.1. and 1.1 kg/m^2^, p=0.002, respectively) while ppFEV1 gains were not different between groups of lung disease severity. P-values refer to Kruskal-Wallis or Mann-U-Whitney inter-group comparisons for continuous variables and chi-square tests for discontinuous variables. ^a^p= 0.039 for <P25 vs. P50 group; ^b^p-value= 0.044 for <P25 vs. >P75 group, ^c^p= 0.005 for <P25 vs. P50 group.BMI = Body mass index; ETI = Elexacaftor/Tezacaftor/Ivacaftor; FU = Follow up; IQR = Inter quartile range; mo. = month; yrs = years; <P25 = baseline ppFEV1 <25^th^ age-specific percentile; P50 = baseline ppFEV1 26^th^ – 74^th^ age-specific percentile; >P75 = baseline ppFEV1 >75^th^ age-specific percentile.

| Characteristics | ≤P25 ppFEV1 (n=55) | P50 ppFEV1 (n=116) | ≥P75 ppFEV1 (n=55) | p-value |
| --- | --- | --- | --- | --- |
| Age at start (yrs), median (IQR) | 23.2 (14.0-37.1) | 22.8 (12.6-30.1) | 20.2 (13.7-29.2) | 0.451 |
| Sex (female, %) | 52.7 | 50.9 | 52.7 | 0.961 |
| CFTR_prior ETI (%) | 49.1 | 46.6 | 38.2 | 0.469 |
| F508del homozygous (%) | 63.6 | 49.1 | 56.4 | 0.195 |
| FEV1% at start, median (IQR) | 31.3 (25.3-57.6) (n=55) | 62.3 (50.1-82.6) (n=116) | 93.7 (79.0-101.2) (n=55) | ------ |
| FEV1% at FU 3 mo, median (IQR) | 43.0 (32.7-75.5) (n=55) | 81.8 (60.1-94.0) (n=116) | 104.1 (89.8-111.8) (n=55) | ------ |
| FEV1% at FU 12 mo, median (IQR) | 37.5 (31.2-61.8) (n=45) | 69.4 (56.2-89.1) (n=92) | 100.3 (86.8-108.2) (n=45) | ------ |
| ∆ FEV1% _(FU 3 mo. - start)_, median (IQR) | 8.1 (4.4-19.1) (n=55) | 10.9 (5.7-19.9) (n=116) | 9.3 (3.4-15.1) (n=55) | 0.209 |
| ∆ FEV1% _(FU 12 mo. - start)_, median (IQR) | 9.0 (4.6-14.2) (n=45) | 10.5 (5.6-22.0) (n=92) | 9.2 (5.9-14.2) (n=45) | 0.258 |
| BMI (kg/m^2^) at start, median (IQR) | 19.1 (16.8-21.4) (n=55) | 19.3 (17.0-21.2) (n=116) | 20.2 (18.0-22.2) (n=55) | 0.041 |
| BMI (kg/m^2^) at FU 3 mo, median (IQR) | 20.1 (18.4-23.6) (n=55) | 20.2 (17.7-22.6) (n=116) | 21.5 (18.4-23.2) (n=55) | 0.121 |
| BMI (kg/m^2^) at FU 12 mo, median (IQR) | 21.3 (19.2-24.7) (n=45) | 21.4 (19.4-23.9) (n=92) | 22.0 (20.6-25.6) (n=45) | 0.261 |
| ∆ BMI (kg/m^2^) _(FU 3 mo. – start)_, median (IQR) | 1.2 (0.6-2.1) **^a, b^** (n=55) | 0.7 (0.0-1.4) **^a^** (n=116) | 0.7 (0.0-1.3) **^b^** (n=55) | 0.001 |
| ∆ BMI (kg/m^2^) _(FU 12 mo. – start)_, median (IQR) | 2.2 (0.6-3.5) **^c^** (n=45) | 1.1 (0.3-2.6) **^c^** (n=92) | 1.1 (0.4-2.3) (n=45) | 0.022 |
