# Supplementary figures and images for: Spirometric and anthropometric improvements in response to elexacaftor/tezacaftor/ivacaftor depending on age and lung disease severity
Source: Front Pharmacol. 2023 Jul 4;14:1171544. doi: 10.3389/fphar.2023.1171544 (PMC10352657; doi:10.3389/fphar.2023.1171544)

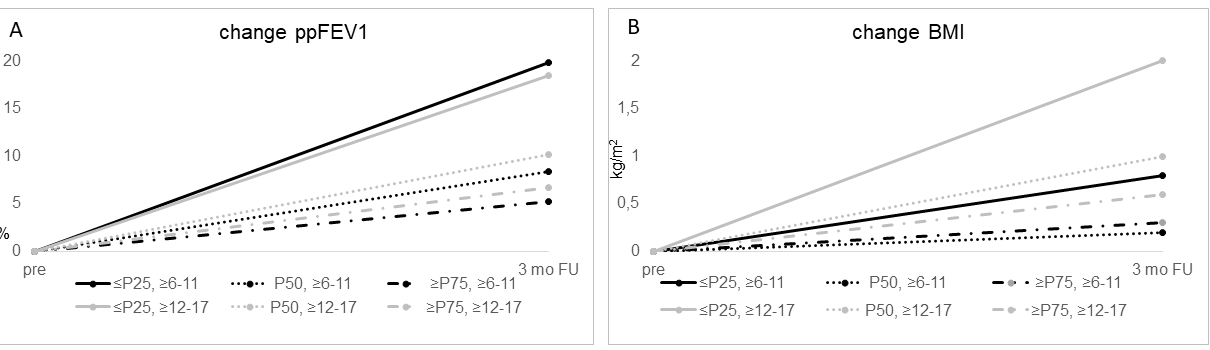

Supplement: Supplementary file 2 [file Image1.TIF]
